# Metabolome and transcriptome analyses provide new insights into the mechanisms underlying the enhancement of medicinal component content in the roots of *Acanthopanax senticosus* (Rupr. et Maxim.) Harms through foliar application of zinc fertilizer

**DOI:** 10.3389/fgene.2023.1259674

**Published:** 2023-11-17

**Authors:** Tongze Sun, Jialin Sun, Yuli Liu, Yanjun Ren, Yifeng Li, Chang Shi, Alyaa Nasr, Zhonghua Tang, Ann Abozeid

**Affiliations:** ^1^ College of Chemistry, Chemical Engineering and Resource Utilization, Northeast Forestry University, Harbin, China; ^2^ Biological Science and Technology Department, Heilongjiang Vocational College for Nationalities, Harbin, China; ^3^ College of Life Science, Kim Il Sung University, Pyongyang, Democratic People’s Republic of Korea; ^4^ Botany and Microbiology Department, Faculty of Science, Menoufia University, Shebeen El-Kom, Egypt

**Keywords:** *Acanthopanax senticosus*, zinc (Zn), foliar fertilization, metabolome, transcriptome

## Abstract

*Acanthopanax senticosus* (Rupr. et Maxim.) Harms is a perennial shrub of the *Acanthopanax* genus in the Araliaceae family and has a high medicinal value. The application of zinc fertilizer can improve the yield and quality of medicinal materials. However, there are limited reports on approaches to increase the content of medicinal components in *A. senticosus*, hindering the improvement of its medicinal quality. In this study, *A. senticosus* was treated with 0.1% (LZn) and 0.4% (HZn) zinc sprayed on the leaf surface. The effects of zinc treatment on the medicinal components in the roots of *A. senticosus* were analyzed by comprehensive metabolomics and transcriptomics analyses. A total of 316 metabolites were detected, with a prevailing occurrence of terpenoids and phenylpropanoids. We identified metabolites related to the medicinal components that were upregulated after Zn treatment, including 43 terpenoids, 19 phenylpropanoids, eight phenols, and three flavonoids. Combining differential gene expression and K-means analysis, we found 95, 65, and 25 upregulated genes related to phenylpropanoid biosynthesis, terpenoid biosynthesis, and flavonoid biosynthesis, respectively. Under different concentrations of Zn treatment, the upregulated metabolite biosynthesis-related genes and differentially expressed transcription factors varied. Pearson correlation network analysis revealed significant correlations among terpenoids, phenylpropanoids, flavonoids biosynthetic genes, and several transcription factors (ERFs, WRKYs, bHLHs, NACs, and MYBs). This study lays the foundation for understanding the metabolic processes in response to varying levels of zinc foliar spray and provides a theoretical basis for enhancing the efficiency of zinc fertilizer utilization in *A. senticosus*.

## 1 Introduction

*Acanthopanax senticosus*, a perennial shrub of the *Acanthopanax* genus in the Araliaceae family, is renowned for its high medicinal value (Sithisarn and Jarikasem, 2009; Huang et al., 2011). Its chemical constituents primarily include phenylpropanoids, flavonoids, terpenoids, lignans, sterols, caffeoylquinic acid derivatives, and fatty acids (Shi et al., 2019; Li et al., 2021). *A. senticosus* has demonstrated various pharmacological effects such as cardioprotection, hypoglycemic activity, hepatoprotection, immunomodulation, neuroprotection, anti-fatigue activity, anti-cancer activity, anti-inflammatory activity, and antioxidant activity (Liang et al., 2010; Bai et al., 2011; Huang et al., 2011; Jiang and Wang, 2015; Kim et al., 2015; Zhang et al., 2015; Zhang et al., 2021).

Zinc (Zn) is a vital trace element for plant growth and development, and it is essential for high yield and quality (Bukhari et al., 2021; Semida et al., 2021). Zn deficiency results in stunted growth as the main symptom in plants (Mattiello et al., 2015). Foliar Zn application can significantly enhance crop quality and yield (Joy et al., 2015; Ahmed et al., 2021). In most studies of medicinal plant fertilization, appropriate fertilization can increase both the yield and quality of medicinal materials and enhance secondary metabolite content. Conversely, inappropriate fertilization can create a stressful environment that negatively impacts medicinal plant growth and quality (Ma et al., 2018; Sun et al., 2021). Foliar fertilization, a widely used agricultural cultivation measure, involves the active absorption of nutrients into leaf interiors (Haslett, 2001). This method is more environmentally friendly than soil application of trace elements, which may produce toxicity (Fernandez and Brown, 2013). Foliar fertilization advantages include low dosage, rapid absorption, minimal pollution, and evident effects (Razzaq et al., 2013; Davarpanah et al., 2016). Zn also maintains the structure of certain protein types, such as transcription factors (TFs) (Xie et al., 2020). Additionally, Zn plays an important role in various physiological functions in plants, such as hormone regulation and signal transduction through mitogen-activated protein kinases (Bhantana et al., 2021; Kaur and Garg, 2021; Ahmad et al., 2022).

Foliar nutrient sprays may vary in their effectiveness depending on their translocation to other plant organs. After foliar application, Zn is taken up by the leaf epidermis, remobilized, and transported to other organs via the phloem (Fernandez and Eichert, 2009; White and Broadley, 2011). However, the remobilization of foliar Zn, from either soil or foliar sources, is influenced by factors such as plant species and genotypes, phenological stage, application method, and environmental conditions (Kutman et al., 2010; Impa et al., 2013). Zn is classified as intermediate or conditionally mobile, but it can still translocate to other organs in many plants following foliar fertilization. This translocation depends on factors such as plant nutritional status, species and variety, or plant phenological state (Wu et al., 2010; Erenoglu et al., 2011; Hegelund et al., 2012).

Currently, transcriptomic and metabolomic analyses have been jointly applied to various medicinal plants, including *Bletilla striata* (Zou et al., 2021), *Dendrobium officinale* (Zhang et al., 2021), and *Ganoderma lucidum* (Meng et al., 2022). Huang et al. (2022) employed a combined transcriptomic and metabolomic analysis to examine changes in flavonoid content and the expression of related biosynthetic pathway genes in Fagopyrum cymosum rhizomes. They uncovered a catechin-related network involving interactions among four metabolites and 14 genes. Liu et al. (2022) discovered that *Astragalus membranaceus* adapted to saline–alkali stress by upregulating flavonoid biosynthesis through a combined transcriptomic and metabolomic analysis, thereby enhancing its medicinal value. However, insufficient reports exist on the metabolome and transcriptome analyses of *A. senticosus*.

In this study, we integrated metabolomic and transcriptomic analyses to investigate changes in medicinal component content and differentially expressed genes (DEGs) in *A. senticosus* roots following foliar application of Zn fertilizer at varying concentrations. By conducting correlation analysis to identify relationships among major differential metabolites, biosynthesis-related genes, and TFs, we provide a reference for enhancing *A. senticosus*’ medicinal quality through Zn fertilization.

## 2 Materials and methods

### 2.1 Plant materials

The test material consisted of 2-year-old *A. senticosus* plants grown at a cultivation base in Hulan District, Harbin City, Heilongjiang Province, China (N45°52′, E126°36′). The field experiment included a control group with no fertilization and only water spray (CK), a second group with foliar application of a low concentration of 0.1% ZnSO_4_·7H_2_O (LZn), and a third group sprayed with a high concentration of 0.4% ZnSO_4_·7H_2_O (HZn). Fertilization was applied on June 7th, June 19th, and July 1st between 4 p.m. and 5 p.m. Each seedling received approximately 25 mL of fertilizer spray, covering both leaf surfaces. On October 25th, roots from plants in different treatments were collected and rinsed with clean water followed by distilled water before being frozen in liquid nitrogen and stored at −80°C for future use. For the metabolome analysis, six biological replicates were performed for each treatment, while the transcriptome analysis had three biological replicates.

### 2.2 Metabolite extraction

The freeze–dried samples were crushed using a mixer mill for 30 s at a frequency of 60 Hz. Then, 100 mg of the sample was added to a solution containing extracted material dissolved in 80% methanol and contained an internal standard of 10 μg/mL. After vortexing for 30 s, the samples were homogenized at a frequency of 45 Hz for 4 minutes and sonicated for 1 hour in an ice-water bath. After being placed in −40°C for 1 hour, the samples were centrifuged at a speed of 12,000 rpm (RCF = 13,800 (×g), R = 8.6 cm) for 15 minutes at 4°C. The supernatant was carefully filtered through a microporous membrane with a pore size of 0.22 μm, and then, 10 μL was taken from each sample and pooled as quality control samples. The samples were stored at −80°C until ultra-high-performance liquid chromatography–mass spectrometry (UHPLC–MS) analysis.

### 2.3 Liquid chromatography–tandem mass spectrometry conditions

An ultra-high-performance liquid chromatography system (Vanquish, Thermo Fisher Scientific) equipped with a Waters UPLC BEH C18 column (1.7 μm 2.1*100 mm) was used for liquid chromatography–tandem mass spectrometry (LC–MS/MS) analysis. The sample injection volume was 5 μL, and the flow rate was 0.5 mL/min. The mobile phase consisted of 0.1% formic acid in water (A) and 0.1% formic acid in acetonitrile (B). The multistep linear elution gradient program was as follows: 85% A for 0–11 min, 25% A for 11–12 min, 2% A for 12–14 min, 2% A for 14–14.1 min, 85% A for 14.1–15 min, and 85% A for 15–16 min.

LC–MS/MS data acquisition was based on the information-dependent acquisition (IDA) mode, and Xcalibur software coupled with a Q Exactive Focus mass spectrometer was used. During each acquisition cycle, the mass range was from 100 to 1,500, and the top three of every cycle were screened, and the corresponding MS/MS data were further acquired. The sheath gas flow rate was set at 45 Arb, the auxiliary gas flow rate was set at 15 Arb, and the capillary temperature was adjusted at 400°C. The full MS resolution was set at 70,000, and the MS/MS resolution was set at 17,500. Collision energy was set at 15/30/45 in the normalized collision energy (NCE) mode, and spray voltage was attuned at +4.0 kV (positive) or −4.0 kV (negative).

### 2.4 Identification and exploration of differentially accumulated metabolites

The raw mass spectrometry data were imported using XCMS software and underwent retention time correction, peak identification, extraction, integration, and alignment. The peaks containing MS/MS data were identified using a secondary mass spectrometry database built by Shanghai Baiqu Biomedical Technology Co., Ltd., along with corresponding fragmentation matching methods. Variable importance in projection (VIP) values were extracted from the orthogonal partial least squares discriminant analysis (OPLS-DA) results to determine the differentially accumulated metabolites (DAMs) between groups with VIP ≥1 and absolute *p*-value ≤0.05.

### 2.5 RNA-seq

The TRIzol reagent (Invitrogen) was used to extract total RNA from tissue samples, and DNase I (TaKara) was used to remove genomic DNA. To monitor RNA degradation and contamination, 1% agarose gels were run. RNA purity was checked using a NanoPhotometer^®^ spectrophotometer (IMPLEN, CA, United States), and RNA concentration was measured with a Qubit^®^ RNA Assay Kit in a Qubit^®^2.0 Flurometer (Life Technologies, CA, United States). RNA integrity was assessed with an RNA Nano 6000 Assay Kit of the Bioanalyzer 2100 system (Agilent Technologies, CA, United States).

RNA sample preparation was performed using 1 µg RNA per sample. The NEBNext^®^ UltraTM RNA Library Prep Kit for Illumina^®^ (NEB, United States) was used to generate sequencing libraries according to the manufacturer’s recommendations. Index codes were added to allocate sequences to each sample. Poly-T oligo-attached magnetic beads were used to purify mRNA from total RNA. Fragmentation was performed using divalent cations under elevated temperature in NEBNext First Strand Synthesis Reaction Buffer (5X). First strand cDNA was synthesized using a random hexamer primer and M-MuLV Reverse Transcriptase (RNase H-). Second strand cDNA synthesis was performed using DNA Polymerase I and RNase H. Remaining overhangs were converted into blunt ends using exonuclease/polymerase activities. After adenylation of 3′ ends of DNA fragments, NEBNext Adaptor with a hairpin loop structure was ligated to prepare for hybridization. The library fragments were purified with the AMPure XP system (Beckman Coulter, Beverly, United States) to select cDNA fragments of 250–300 bp in length. Size-selected, adaptor-ligated cDNA was treated with 3 µL USER Enzyme (NEB, United States) at 37°C for 15 min followed by 5 min at 95°C before PCR. Finally, PCR products were purified (AMPure XP system), and library quality was assessed on the Agilent Bioanalyzer 2100 system.

The original data were filtered using fastp, mainly to eliminate reads containing adapters. Paired reads were removed if the N content in any sequencing read exceeded 10% of the base number of the reads. Paired reads were also removed if the number of low-quality (Q < = 20) bases contained in any sequencing read exceeded 50% of the bases of the read. All subsequent analyses were based on clean reads. Trinity was used to perform transcriptome assembly. Relevant transcripts were regrouped into gene clusters using Corset (https://github.com/trinityrnaseq/trinityrnaseq). TransDecoder (https://github.com/TransDecoder/TransDecoder/wiki) was used to identify candidate coding regions within transcript sequences generated by *de novo* RNA-Seq transcript assembly using Trinity. Gene function was annotated based on the following databases using diamond or HMMER: Nr, Swiss-Prot, Trembl, KEGG, GO, KOG/COG, and Pfam. Gene expression levels were estimated by RSEM, and FPKM of each gene was calculated based on the gene length.

### 2.6 Differential expression, gene mining, and enrichment analysis

DESeq2 was used to analyze differential expression between the two groups. A *p*-value less than 0.05 and |log2foldchange|≥1 were used as the threshold for significant differential expression. We used Goatools (https://github.com/tanghaibao/Goatools) and KOBAS (http://kobas.cbi.pku.edu.cn/home.do) to perform GO functional enrichment and KEGG pathway analysis for the DEGs to understand their functions. GO terms and metabolic pathways with a Bonferroni-corrected *p*-value less than 0.05 were considered as significantly enriched by DEGs.

### 2.7 Correlation network analysis

Principal component analysis (PCA) and Pearson correlation analysis were performed using R’s built-in functions. Subsequently, significant correlation networks (*p* < 0.05) between biosynthesis genes and metabolites and between biosynthesis genes and TFs were visualized using Cytoscape 3.9 (Cline et al., 2007).

## 3 Results

### 3.1 Metabolomic analysis

**FIGURE 1:**
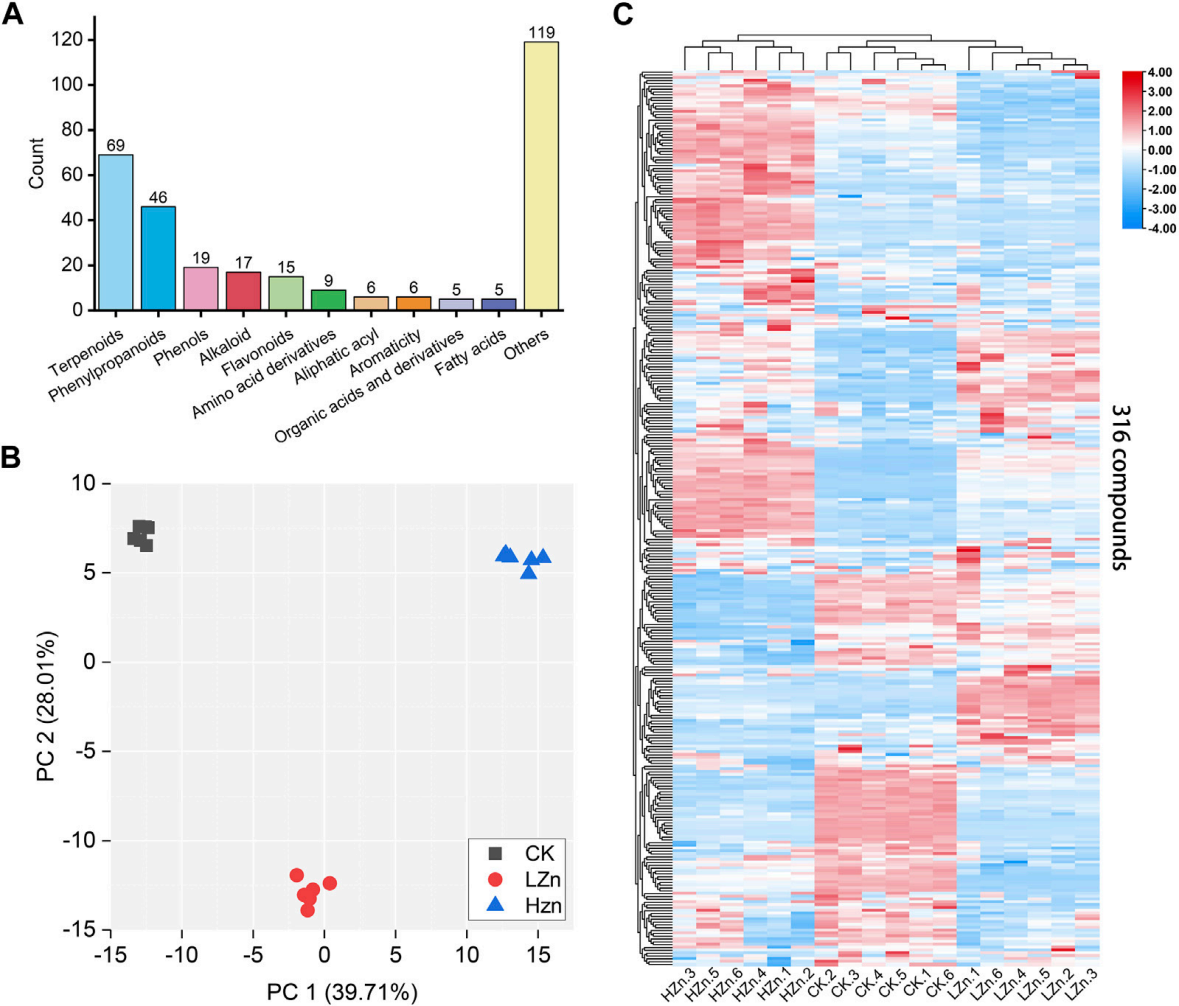
Metabolomics analysis based on LC–MS/MS measurement. **(A)** Types and quantities of metabolites, **(B)** PCA score plot, and **(C)** hierarchical clustering heatmap of 316 metabolites in three sample groups.

A total of 316 metabolites were identified in the samples; among them, terpenoids were the most abundant, accounting for 21.8% of the total, followed by phenylpropanoids at 14.6% (Supplementary Table S1). Phenols, alkaloids, flavonoids, amino acid derivatives, fatty acyls, aromatic compounds, organic acids and their derivatives, fatty acids, and other compounds accounted for 6.0%, 5.4%, 4.7%, 2.8%, 1.9%, 1.9%, 1.6%, 1.6%, and 37.7%, respectively (Figure 1A). To compare the metabolic differences among HZn, LZn, and CK, PCA was performed on the metabolomics dataset. In the PCA score plot, the contribution rates of PC1 and PC2 were 39.71% and 28.01%, respectively. Each sample group was clustered together, indicating good repeatability of the samples. The samples between different groups were clearly alienated, indicating significant differences between the metabolomes (Figure 1B). Hierarchical clustering heatmap analysis showed that the replicates of the samples were assembled together, further indicating good data repeatability. In addition, the color differences of the 316 metabolites between different samples were obvious, demonstrating significant differences in metabolites between different samples (Figure 1C).

**FIGURE 2:**
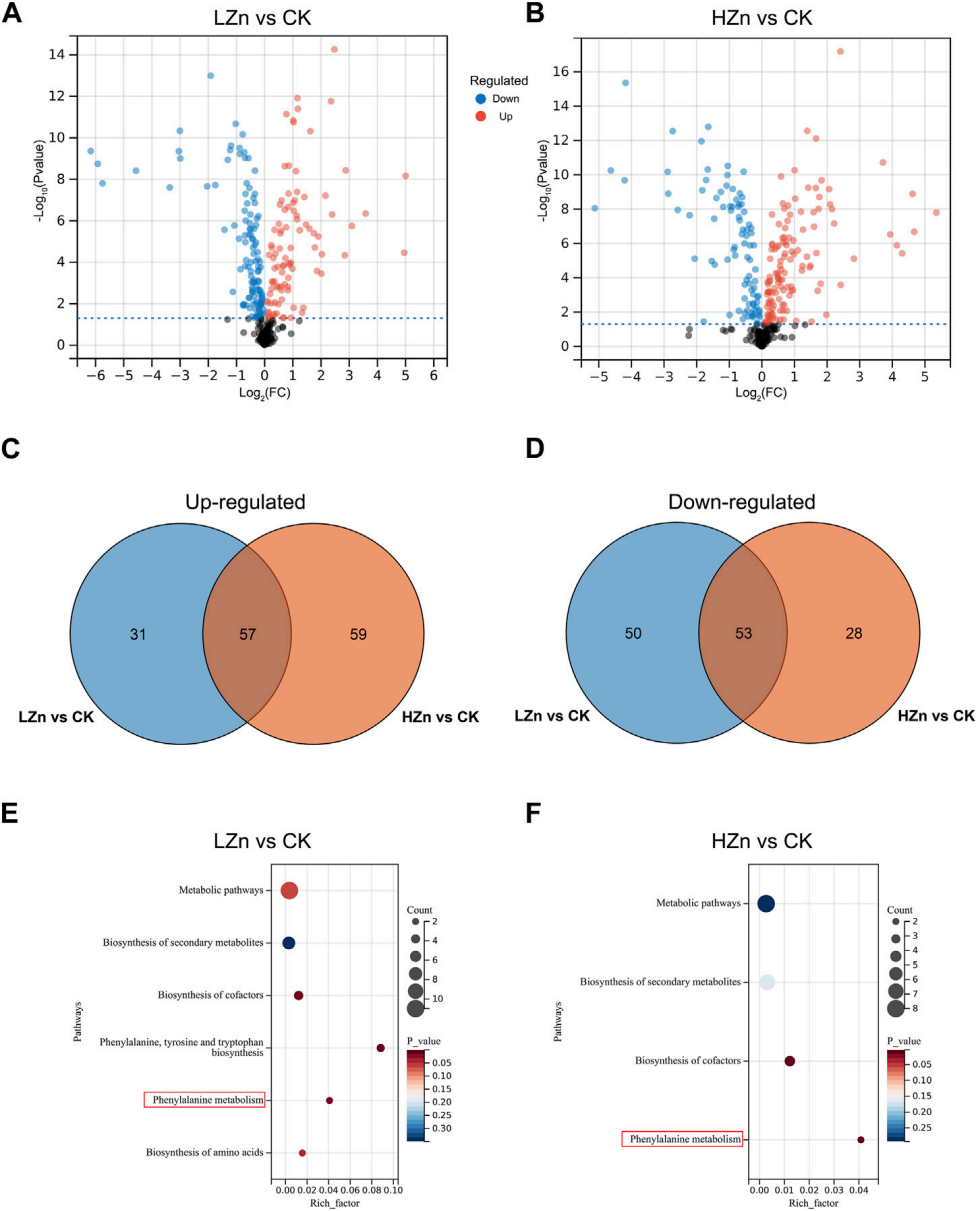
Identification and enrichment analysis of DAMs. Identification of DAMs in LZn vs. CK **(A)** and HZn vs. CK **(B)** comparisons. Venn diagram of the upregulated **(C)** and downregulated **(D)** DAMs between LZn vs. CK and HZn vs. CK. KEGG enrichment analysis in LZn vs. CK **(E)** and HZn vs. CK **(F)** comparisons.

DAMs were screened using the criteria of VIP >1 and *p*-value <0.05. A total of 191 DAMs were identified in LZn vs. CK, including 88 upregulated metabolites and 103 downregulated metabolites (Figure 2, Supplementary Table S2). These mainly comprised 53 terpenoids, 25 phenylpropanoids, 12 alkaloids, 10 phenols, seven flavonoids, and 84 other metabolites. Compared with CK, these upregulated compounds in LZn treatment increased by at least 5.86%. Among them, the highest fold changes were observed for isoimperatorin (phenylpropanoid), fraxetin (phenylpropanoid), and flavanone base + 3O, 1Prenyl (flavonoid), which increased by more than 29.98 times compared with CK. In HZn vs. CK, a total of 197 DAMs were identified, including 47 terpenoids, 25 phenylpropanoids, 13 alkaloids, nine phenols, six flavonoids, and 89 other compounds. These upregulated compounds in HZn treatment increased by at least 11.71% compared with CK. The highest fold changes were observed for flavanone base + 3O, 1Prenyl (flavonoid), fraxetin (phenylpropanoid), and cortodoxone (terpenoid), which increased by more than 24.47 times compared with CK. Venn diagram analysis showed that among the upregulated metabolites, HZn vs. CK and LZn vs. CK had 59 and 31 upregulated metabolites, respectively, with a total of 57 shared between them. Among the downregulated metabolites, HZn vs. CK and LZn vs. CK had 28 and 50 downregulated metabolites, respectively, with a total of 53 shared between them. KEGG enrichment analysis revealed that the DAMs of LZn vs. CK were mainly significantly enriched in metabolic pathways such as biosynthesis of secondary metabolites, biosynthesis of cofactors, phenylalanine, tyrosine, and tryptophan biosynthesis, phenylalanine metabolism, and biosynthesis of amino acids. On the contrary, the focal differential metabolites of HZn vs. CK were considerably enriched in metabolic pathways such as biosynthesis of secondary metabolites, biosynthesis of cofactors, and phenylalanine metabolism.

**FIGURE 3:**
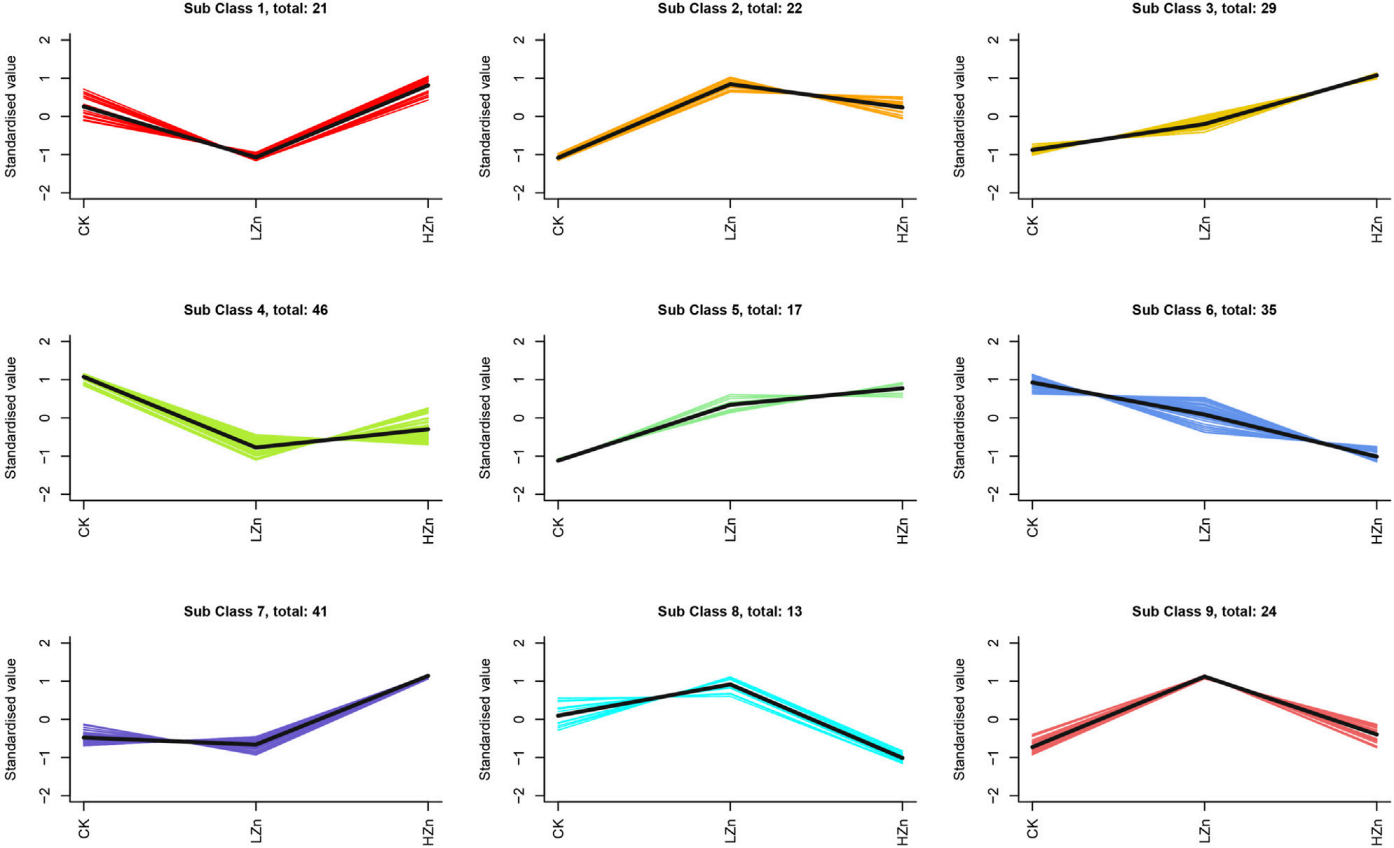
K-means clustering analysis of 228 DAMs.

**FIGURE 4:**
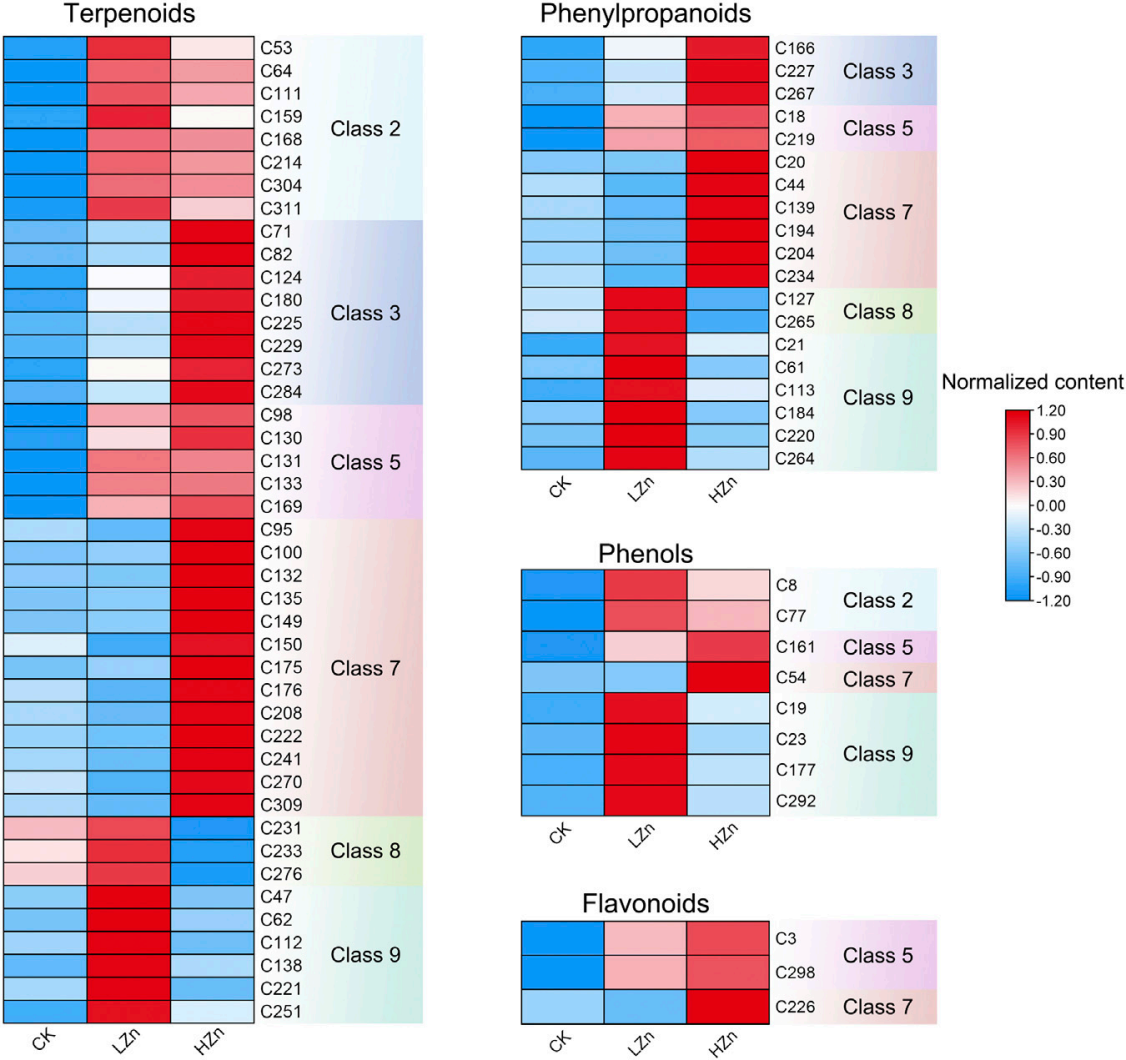
Terpenoids, phenylpropanoids, phennols, and flavonoids with upregulated content in the *A. senticosus* root after foliar application of Zn fertilizer. The classes are derived from the results of the K-means analysis.

K-means clustering analysis proposed that 228 differential metabolites could be divided into nine classes (Figure 3), numbered as 1–9, and comprised 21, 22, 29, 46, 17, 35, 41, 13, and 24 metabolites, respectively (Supplementary Table S2). Notably, the metabolite content was increased in classes 2, 3, 5, 7, 8, and 9 after Zn treatment. Of these, classes 2 and 5 had a significant metabolite increment in both LZn and HZn treatment groups. Based on the differential metabolite analysis and K-means analysis, we identified medicinally related components of *A. senticosus* that amplified after Zn treatment, including 43 terpenoids, 19 phenylpropanoids, eight phenols, and three flavonoids (Figure 4). The analysis of the 43 terpenoid compounds revealed that eight compounds belong to class 2, eight to class 3, five to class 5, 13 to class 7, three to class 8, and the remaining six to class 9. Similarly, among the 19 phenylpropanoids studied, class distribution was as follows: three in class 3, two in class 2, six in class 7, two in class 8, and the remaining six in class 9. In the case of the eight phenols analyzed, two were found to belong to class 2, one to class 5, one to class 7, and the remaining four to class 9. Last, of the three flavonoids examined, two were classified into class 5 and one into class 7.

### 3.2 Transcriptome analysis

**FIGURE 5:**
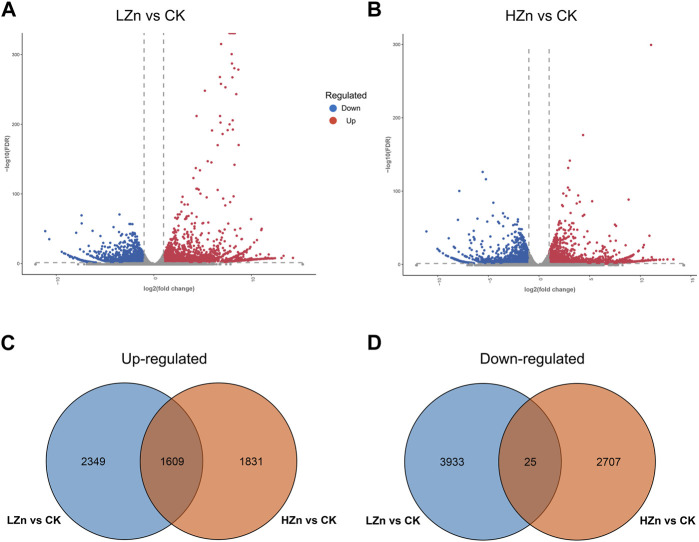
Identification of DEGs. **(A)** DEGs between LZn and CK. **(B)** DEGs between HZn and CK. Venn diagram of the upregulated **(C)** and downregulated **(D)** DEGs between LZn vs. CK and HZn vs. CK.

RNA-seq sequencing yielded 53.95 GB of raw data (Supplementary Table S3). After quality control filtering, 49.62 GB of valid data were obtained (Supplementary Table S4). In the valid data, the Q20 value exceeded 97%, and the Q30 value exceeded 92% (Supplementary Table S4). The GC content ranged from 43.27% to 49.11% (Supplementary Table S4). Gene annotation results showed that a total of 110,322 genes were successfully annotated (Supplementary Table S5). Of these, 54,526; 37,961; 45,013; 45,947; 59,707; and 56,103 genes were annotated in the GO, KEGG, Pfam, Swiss-Prot, and NR databases (Supplementary Table S6). As shown in the volcano plot in Figure 5, red color represents significantly upregulated genes, blue represents significantly downregulated genes, and gray represents genes with no significant difference. In LZn vs. CK, a total of 6,696 differentially expressed genes were obtained, including 3,958 upregulated and 2738 downregulated (Figure 5A). In HZn vs. CK, a total of 6,172 differentially expressed genes were obtained, including 3,440 upregulated and 2,732 downregulated (Figure 5B). Venn diagram analysis showed that among the upregulated differentially expressed genes, HZn vs. CK and LZn vs. CK had 1831 and 2,349 genes, respectively, with a total of 1,609 upregulated genes shared between them (Figure 5C). Among the downregulated differentially expressed genes, HZn vs. CK and LZn vs. CK had 2,707 and 3,933 genes, respectively, with a total of 25 downregulated genes shared between them (Figure 5D).

**FIGURE 6:**
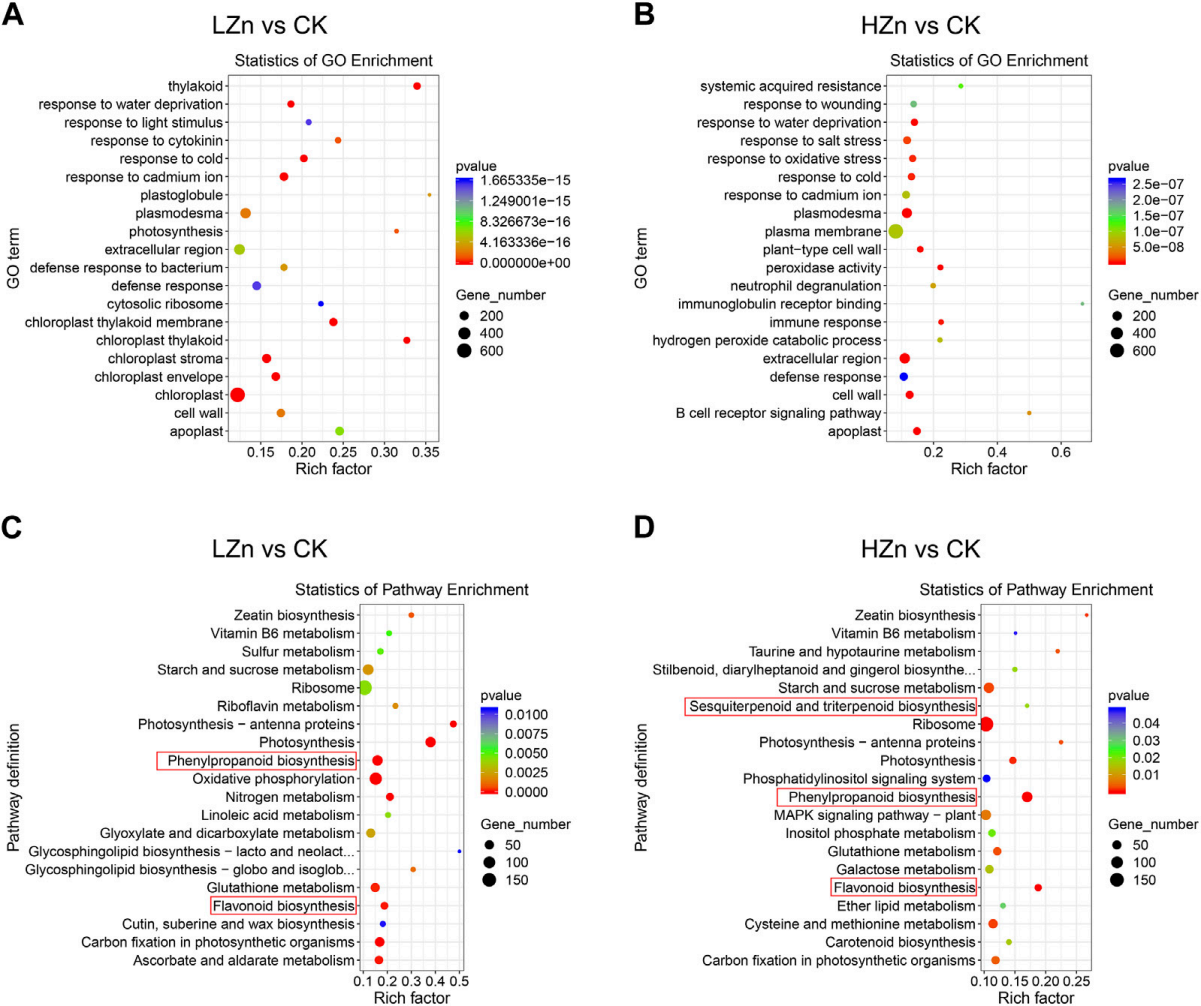
GO and KEGG enrichment analysis of differentially expressed genes. GO enrichment analysis in LZn vs. CK **(A)** and HZn vs. CK **(B)** comparisons. KEGG enrichment analysis in LZn vs. CK **(C)** and HZn vs. CK **(D)** comparisons.

GO functional enrichment analysis was performed on the differentially expressed genes. Figure 6 shows that in LZn vs. CK, the top five significantly enriched pathways for differentially expressed genes were chloroplast stroma, thylakoid, chloroplast thylakoid membrane, response to cold, and chloroplast (Figure 6A). In HZn vs. CK, the top five significantly enriched pathways for differentially expressed genes were plasmodesma, extracellular region, apoplast, peroxidase activity, and cell wall. According to the KEGG pathway enrichment analysis, the top 20 most significantly enriched metabolic pathways indicating that genes related to the metabolic pathways of medicinal components changed significantly after Zn treatment (Figure 6B). In LZn vs. CK, the significantly enriched pathways related to medicinal metabolites were phenylpropanoid biosynthesis and flavonoid biosynthesis (Figure 6C). In case of HZn vs. CK, phenylpropanoid biosynthesis, flavonoid biosynthesis, and sesquiterpenoid and triterpenoid biosynthesis were spotted as the substantial enriched pathways related to medicinal metabolites (Figure 6D). We also identified differentially expressed TFs. In LZn vs. CK, a total of 192 differentially expressed TFs were identified, including 110 upregulated and 82 downregulated (Supplementary Figure S1, Supplementary Table S7). In HZn vs. CK, a total of 164 differentially expressed TFs were recognized, including 97 upregulated and 67 downregulated (Supplementary Figure S2, Supplementary Table S7). Furthermore, a distinct pattern was observed in the upregulation of genes under different zinc treatments. Specifically, in the DEGs of LZn vs. CK comparison, but not in the DEGs of HZn vs. CK comparison, four flavonoid biosynthesis-related genes, five phenylpropanoid biosynthesis-related genes, and 10 terpenoid biosynthesis-related genes were upregulated. Conversely, in the DEGs of HZn vs. CK comparison, but not in the DEGs of LZn vs. CK, two flavonoid biosynthesis-related genes, 22 phenylpropanoid biosynthesis-related genes, and 12 terpenoid biosynthesis-related genes were upregulated. These findings suggest that varying concentrations of zinc treatment can differentially influence the expression of biosynthetic genes.

**FIGURE 7:**
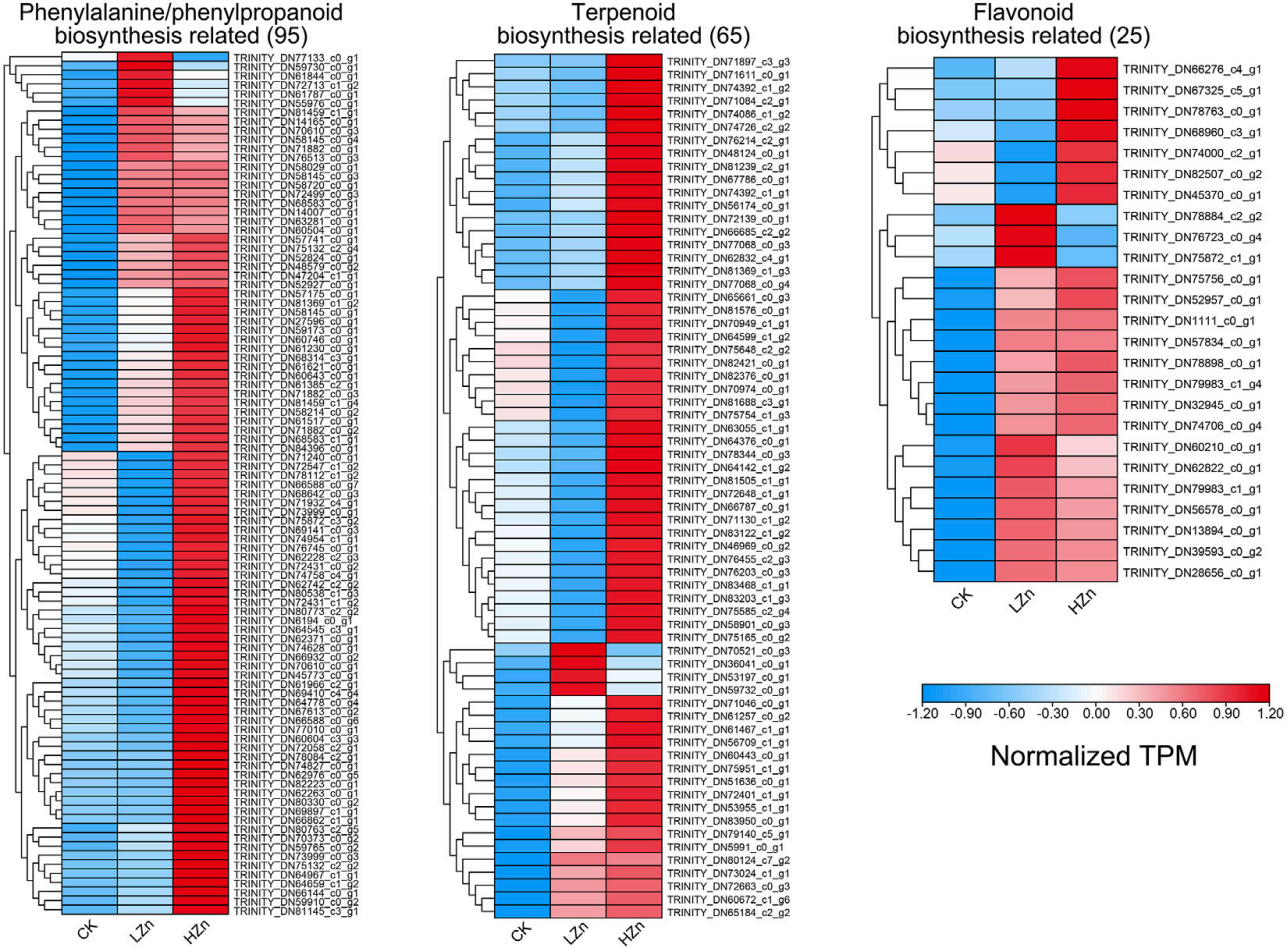
Upregulated genes related to the biosynthesis of important medicinal components in *A. senticosus* after foliar application of Zn fertilizer.

The results of the k-means fuzzy clustering analysis suggested that all differentially expressed genes can be categorized into four classes (Supplementary Figure S3). Class 1 contains 6,102 genes, which exhibited low expression in both CK and LZn and high expression in HZn. Class 2 contains 9,387 genes, whose expression levels decreased after LZn and HZn treatment. Class 3 contains 2596 genes, whose expression levels increased after LZn and HZn treatment. Class 4 contains 9,913 genes, which showed high expression in both CK and HZn treatment but feeble expression after LZn treatment. To reveal the reasons for the upregulation of phenylpropanoid, terpenoid, and flavonoid compounds by zinc treatment, we identified 95, 65, and 25 differentially expressed genes related to phenylpropanoid biosynthesis, terpenoid biosynthesis, and flavonoid biosynthesis in classes 1 and 3, respectively (Figure 7, Supplementary Table S8). Consistent with existing reports, multiple transcription factor families such as ERF, bHLH, MYB, NAC, WRKY, and bZIP have transcriptional regulatory effects on these metabolites. In classes 1 and 3, there are a total of 12 bHLHs, 16 bZIPs, 34 ERFs, 13 MYBs, 14 MYB_related, 13 NACs, and 25 WRKY genes, suggesting that they have potential transcriptional regulatory effects on the biosynthesis of related metabolites (Supplementary Table S9).

### 3.3 Integrated analysis of metabolomics and transcriptomics

**FIGURE 8:**
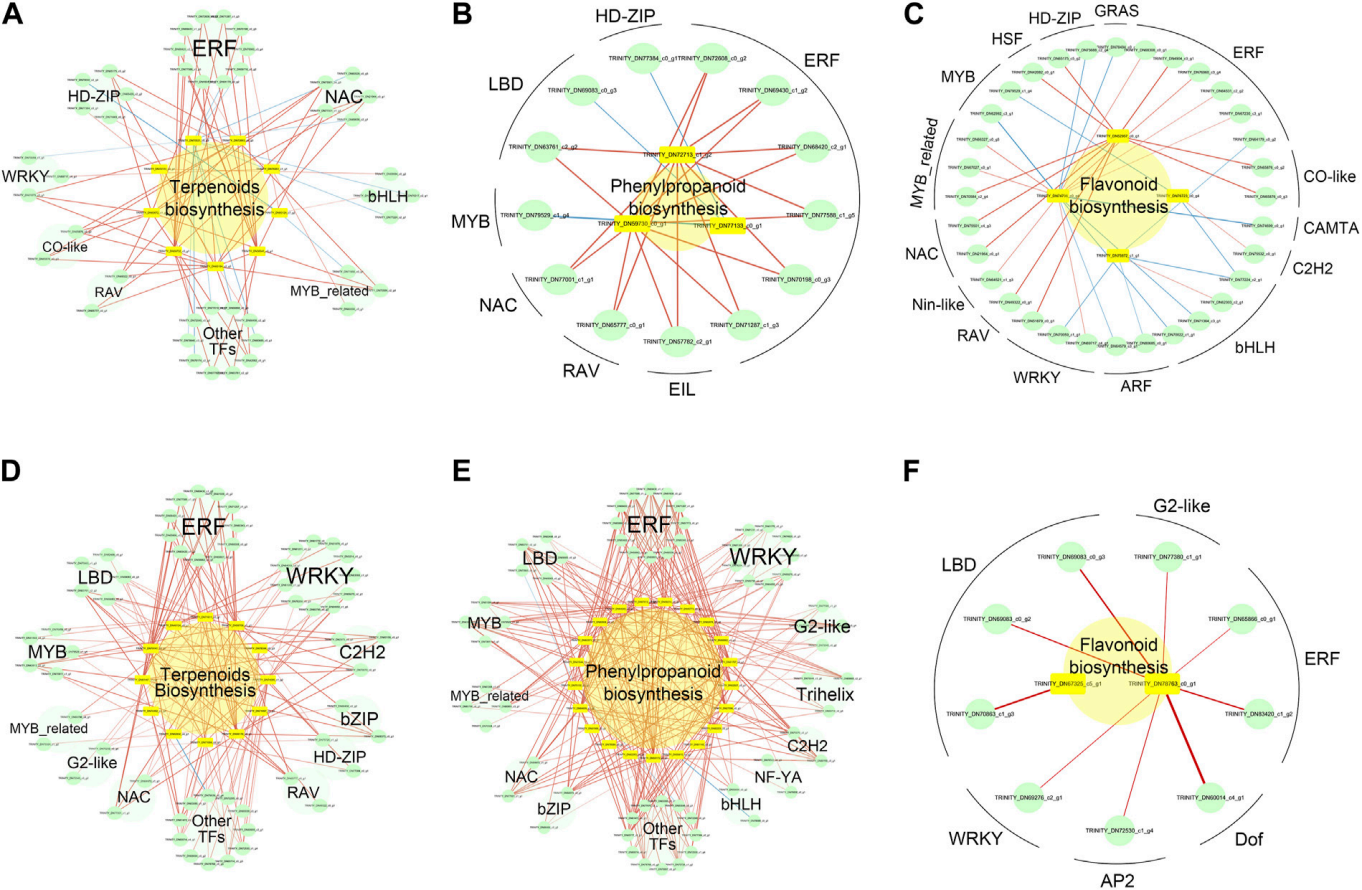
Significant correlation network between key metabolite biosynthesis genes and TFs in *A. senticosus* after foliar application of Zn fertilizer. In the upregulated metabolite biosynthesis, genes between LZn and CK, but not between HZn and CK, correlation networks between terpenoids **(A)**, phenylpropanoids **(B)**, and flavonoids **(C)** biosynthesis key genes and TFs. In the upregulated metabolite biosynthesis, genes between HZn and CK, but not between LZn and CK, correlation networks between terpenoids **(D)**, phenylpropanoids **(E)**, and flavonoids **(F)** biosynthesis key genes and TFs.

To better understand the metabolic regulatory network of *A. senticosus* after treatment with different concentrations of zinc, we performed a Pearson correlation analysis on the differentially expressed genes related to metabolite biosynthesis and TFs (Figure 8). A significant correlation was established between TFs and structural genes when the *p*-value was less than 0.05 and the correlation coefficient |*r|* exceeded 0.9.

In the upregulated metabolite biosynthesis genes between LZn and CK, but not between HZn and CK, 44 transcription factors were identified to have a significant correlation with eight terpenoid biosynthesis-related genes (Figure 8A, Supplementary Table S10). These transcription factors included 10 ERFs, five NACs, five HD-ZIPs, four bHLHs, three WRKYs, three MYB_related, two CO-like, two RAVs, and 10 other types of transcription factors. Among them, 30 transcription factors showed a significant positive correlation with eight terpenoid biosynthesis-related genes, while 14 transcription factors had a significant negative correlation with two terpenoid biosynthesis-related genes. Thirteen transcription factors exhibited a significant positive correlation with three phenylpropanoid biosynthesis-related genes, including six ERFs, one EIL, one RAV, one NAC, one MYB, two LBDs, and one HD-ZIP (Figure 8B). Additionally, 32 transcription factors were significantly correlated with four flavonoid biosynthesis-related genes (Figure 8C). These comprised six ERFs, two CO-like, four bHLHs, two ARFs, three WRKYs, two NACs, three MYB-related, two MYBs, two HD-ZIPs, and six other types of transcription factors.

In the upregulated metabolite biosynthesis genes between HZn and CK, but not between LZn and CK, significant correlations were observed between the expression of metabolite biosynthesis-related genes and various transcription factors (Supplementary Table S11). Specifically, 58 transcription factors were significantly correlated with 12 terpenoid biosynthesis-related genes (Figure 8D). These included 11 ERFs, 11 WRKYs, three C2H2s, two bZIPs, two HD-ZIPs, two RAVs, five LBDs, five MYBs, two MYB_relateds, two G2-likes, two NACs, and 11 other types of transcription factors. Among these transcription factors, only one showed a significant negative correlation with a structural gene, while the rest showed a significant positive correlation. A total of 63 transcription factors were significantly correlated with 12 phenylpropanoid biosynthesis-related genes (Figure 8E). These comprised 12 ERFs, nine WRKYs, four G2-likes, three trihelixes, two C2H2s, two NF-YAs, two bHLHs, two bZIPs, two NACs, four MYBs, four MYB_relateds, five LBDs, and 12 other types of transcription factors. Among these transcription factors, only three showed a significant negative correlation with three structural genes. Nine transcription factors demonstrated a significant positive correlation with two flavonoid biosynthesis-related genes (Figure 8F). These included three LBDs, two ERFs, one G2-like, one WRKY, one AP2, and one Dof.

## 4 Discussion

In this study, we investigated the effect of foliar application of different Zn concentrations on the roots of *A. senticosus*. Our results revealed that foliar application of a certain Zn concentration can significantly increase the content of medicinal components in the roots of *A. senticosus*. Compared with the control, foliar application of different Zn levels markedly enhanced the content of many medicinal metabolites in the roots of *A. senticosus*, including 43 terpenoids, 19 phenylpropanoids, eight phenols, and three flavonoids. In our study, DEG analysis and K-means clustering identified 95 upregulated phenylpropanoid biosynthesis-related genes, 65 terpenoid biosynthesis-related genes, and 25 flavonoid biosynthesis-related genes after Zn treatment. We also observed significant correlations between the expression of metabolite biosynthesis-related genes and various transcription factors in the upregulated metabolite biosynthesis genes between LZn and CK, but not between HZn and CK, or between HZn and CK, but not between LZn and CK. These findings suggest that in *A. senticosus*, foliar application of Zn fertilizer may regulate the expression of various medicinal compound biosynthesis genes by affecting the expression of related transcription factors, thereby increasing the content of medicinal components in the roots.

Our results are consistent with many previous studies. First, foliar application of zinc fertilizer can increase the content of medicinal components in various plants. For example, in *Stevia rebaudiana*, foliar application of zinc sulfate significantly increased its stevioside content (Singh and Dwivedi, 2019). In *Mentha pulegium*, zinc treatment significantly increased the composition of pulegone, cis-isopulegone, a-pinene, sabinene, 1,8-cineole, and borneol in mint essential oil (Lajayer et al., 2016). In *Lavandula stoechas*, foliar application of zinc fertilizer had a substantial effect on its flavonoid and volatile oil contents (Vojodi Mehrabani et al., 2017). Furthermore, in *Melissa officinalis*, foliar application of zinc fertilizer significantly increased the plant contents of flavonoids and phenolic compounds (Yadegari, 2017). In *Panax ginseng*, foliar application of zinc fertilizer considerably improved the root yield and increased its contents of ginsenoside monomer along with other nine saponins (Zhang et al., 2013). Furthermore, trigonelline content was augmented in fenugreek (*Trigonella foenum-graecum* L.) when zinc fertilizer was applied (Tariverdizadeh et al., 2021). These studies all indicate that foliar application of Zn fertilizer can indeed enhance the quality of medicinal plants. Therefore, foliar application of Zn fertilizer can be widely used as a fertilization technique to improve the quality of medicinal components in *A. senticosus* roots.

Additionally, preliminary studies indicated that Zn fertilizer treatments could alter the expression of genes encoding enzymes involved in metabolite synthesis, thereby affecting changes in metabolite content. For example, under zinc stress, the activities and gene expression levels of ascorbate peroxidase, catalase, superoxide dismutase, and peroxidase were upregulated in tea plants (Mukhopadhyay et al., 2013). Furthermore, Zn fertilizer treatment considerably increased the antioxidant potentials (ascorbic acid, reduced glutathione, total phenols, and total flavonoids) in wheat flag leaves and enhanced the expression of two antioxidant enzyme genes, four ascorbate-glutathione cycle genes, and two flavonoid biosynthesis-related genes (Sun et al., 2020). Dong et al. (2021) also found that Zn fertilizer treatment could affect the expression of stress-related resistance genes in different barley cultivars. In our study, DEG analysis and K-means clustering emphasized 95 upregulated phenylpropanoid biosynthesis-related genes, 65 terpenoid biosynthesis-related genes, and 25 flavonoid biosynthesis-related genes after Zn treatment. However, the molecular mechanisms underlying the effects of foliar Zn application on the expression of these medicinal metabolite synthesis enzyme genes are still not very clear. Some studies have shown that plant responses to Zn deficiency are regulated by multiple levels, including transcriptional regulation mediated by F-group bZIP proteins, epigenetic regulation at the level of chromatin, and post-transcriptional regulation mediated by small RNAs and alternative splicing (Zeng et al., 2021). These need to be gradually clarified in future studies.

TFs regulate gene expression, allowing plants to respond to stress and modulate developmental processes (Mitsuda and Ohme-Takagi, 2009). Plants have approximately 58 TF families, and six major TF families (AP2/ERF, bHLH, MYB, NAC, WRKY, and bZIP) are involved in biotic and abiotic stress responses (Ng et al., 2018). The MYB family is the largest TF family in plants, and its members play crucial roles in plant development, stress responses, and secondary metabolite biosynthesis (Wang et al., 2021). Members of the ERF (Huang et al., 2021), NAC (Nuruzzaman et al., 2013; Borchers and Pieler, 2022), WRKY (Chi et al., 2013), bZIP (Zhao et al., 2021), and bHLH (Mao et al., 2017) gene families also contribute essentially in plant stress responses and metabolite biosynthesis. In this study, we identified 34 ERFs, 12 bHLHs, 13 NACs, 14 MYB_relateds, 13 MYBs, 16 bZIPs, and 25 WRKY genes with significantly altered expression levels after Zn treatment (Supplementary Table S9). These genes were also significantly correlated with multiple medicinal metabolite biosynthesis-related genes. Proposing that, these TFs may respond to Zn treatment stimuli and regulate the expression of multiple metabolite biosynthesis genes, thereby participating in the production of related metabolites. Additionally, different Zn concentrations resulted in different upregulated terpenoid, phenylpropanoid, and flavonoid biosynthesis genes and differentially expressed transcription factors, indicating that different Zn concentrations could regulate the biosynthesis of metabolites by affecting specific transcription factors that control the expression of metabolite biosynthesis-related genes. Further research on the transcriptional regulatory mechanisms of these TFs would lay the foundation for elucidating the molecular mechanisms underlying the enhancement of medicinal component accumulation in *A. senticosus* roots by Zn treatment.

## 5 Conclusion

Our research used metabolomics and transcriptomics to examine change patterns in metabolites and genes in *A. senticosus* roots following foliar application of varying levels of zinc. A total of 316 metabolites were detected, including 69 terpenoids, 46 phenylpropanoids, 19 phenols, 17 alkaloids, 15 flavonoids, nine amino acid derivatives, six aliphatic acyls, six aromatics, five organic acids and derivatives, five fatty acids, and 119 other compounds. Differential metabolite analysis and K-means clustering identified metabolites related to the medicinal components of *A. senticosus* that were upregulated after Zn treatment, including 43 terpenoids, 19 phenylpropanoids, eight phenols, and three flavonoids. Transcriptome analysis showed significant changes in the phenylpropanoid and flavonoid biosynthesis pathways after Zn treatment. Differential gene expression analysis and K-means clustering marked a total of 95 phenylpropanoid biosynthesis-related genes, 65 terpenoid biosynthesis-related genes, and 25 flavonoid biosynthesis-related genes. Correlation network analysis revealed significant and positive parallels between three flavonoids and 16 flavonoid biosynthesis genes, 19 phenylpropanoids and 95 phenylpropanoid biosynthesis-related genes, and 43 terpenoids and 66 terpenoid biosynthesis-related genes. Additionally, significant correlations were observed between multiple medicinal component biosynthesis-related genes and some key TFs (ERFs, WRKYs, bHLHs, NACs, and MYBs). This study provides a valuable reference for increasing the medicinal components content in *A. senticosus* roots through Zn fertilizer application.

## Data Availability

The datasets presented in this study can be found in online repositories. The names of the repository/repositories and accession number(s) can be found at: https://www.ncbi.nlm.nih.gov/, PRJNA972895.
