# Supplementary figures and images for: Metabolome and transcriptome analyses provide new insights into the mechanisms underlying the enhancement of medicinal component content in the roots of Acanthopanax senticosus (Rupr. et Maxim.) Harms through foliar application of zinc fertilizer
Source: Front Genet. 2023 Nov 17;14:1259674. doi: 10.3389/fgene.2023.1259674 (PMC10690782; doi:10.3389/fgene.2023.1259674)

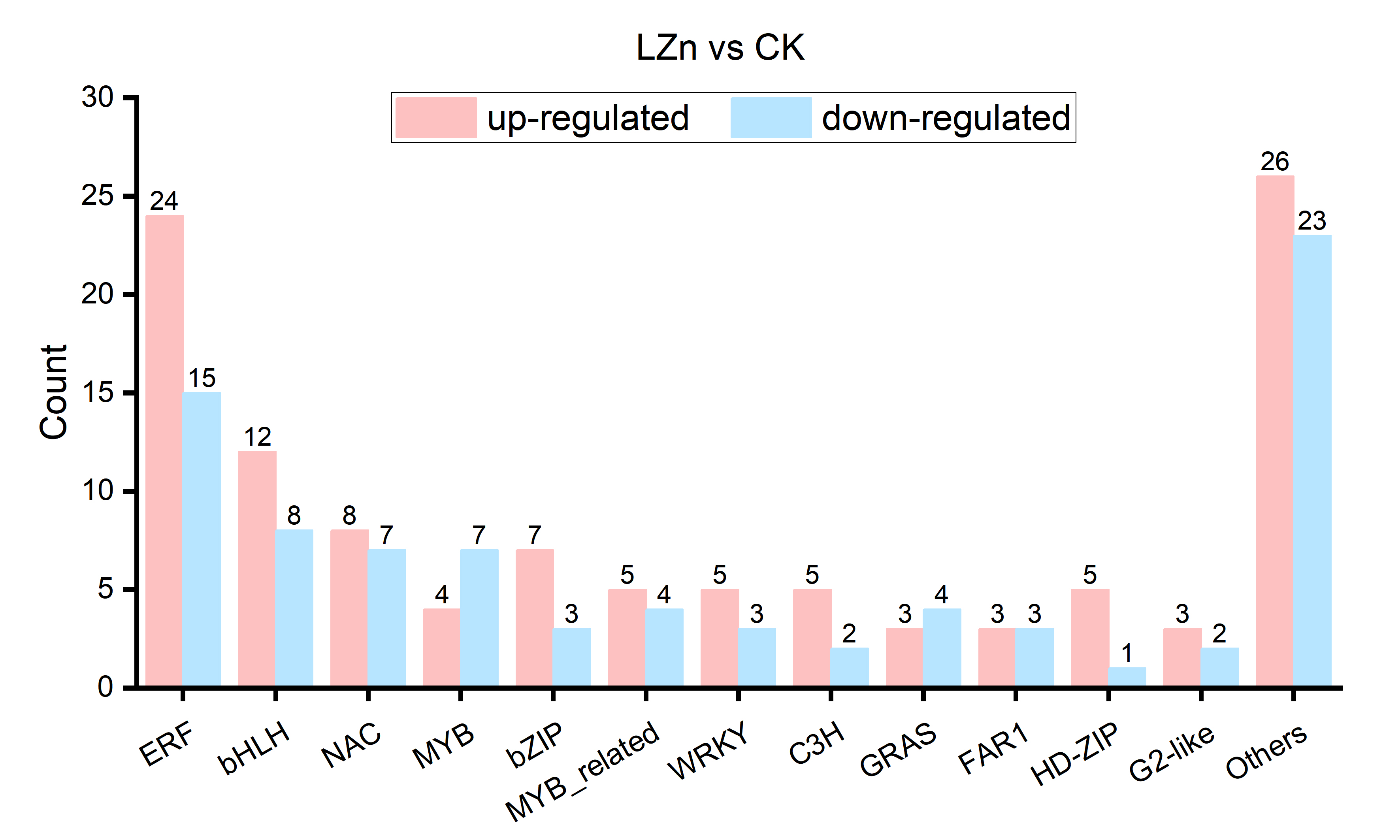

Supplement: Supplementary file 1 [file DataSheet1.ZIP › Supplementary files/Figure S1.jpg]

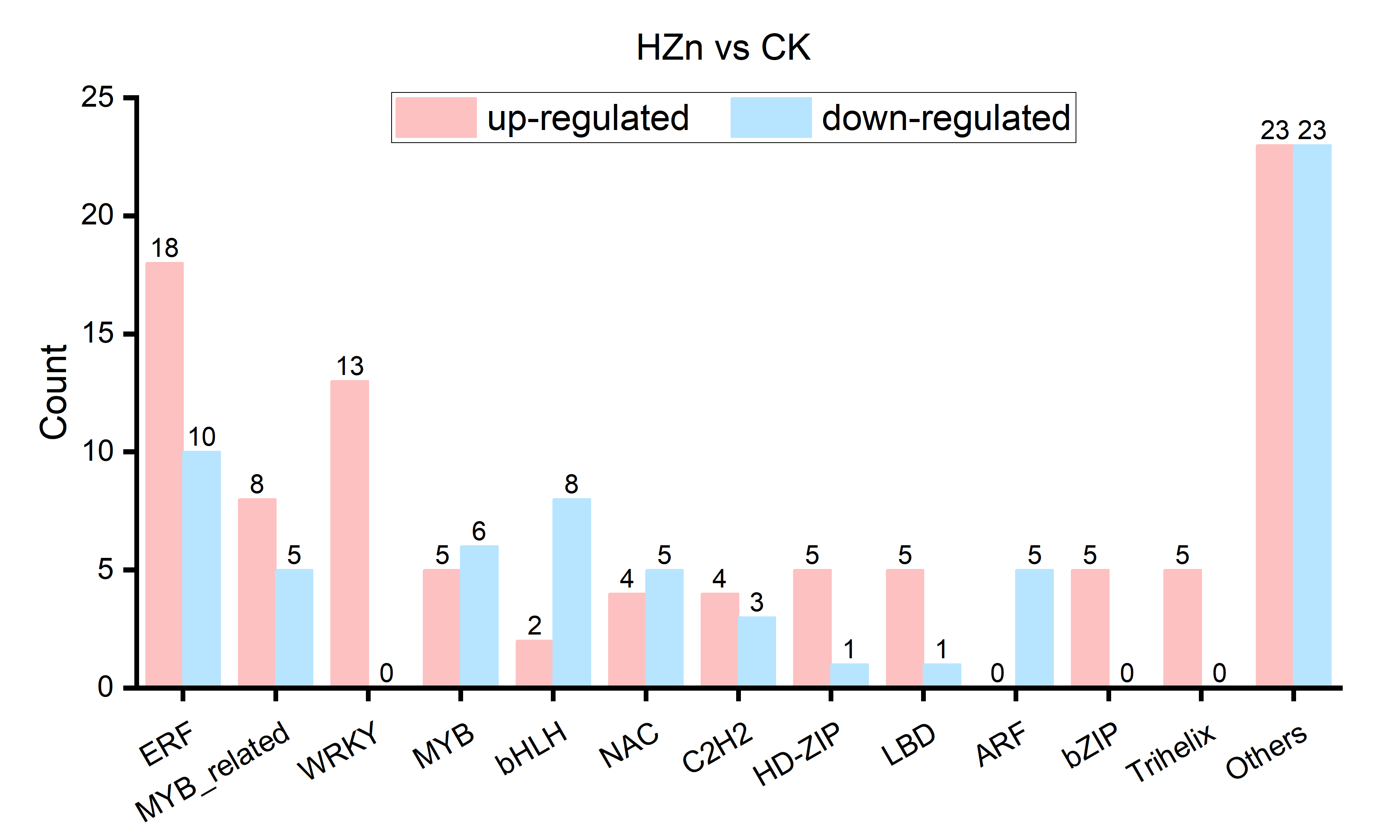

Supplement: Supplementary file 1 [file DataSheet1.ZIP › Supplementary files/Figure S2.jpg]

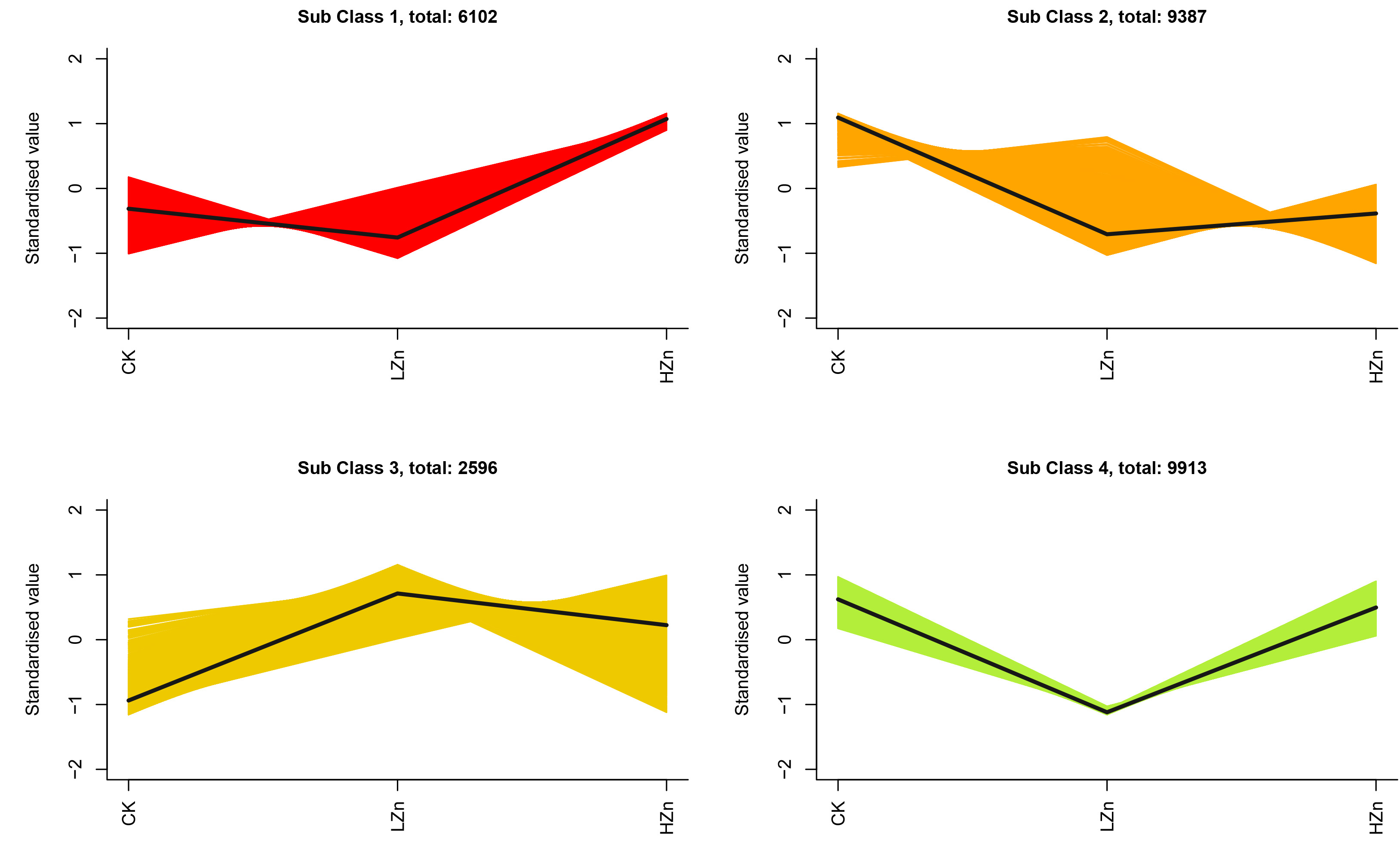

Supplement: Supplementary file 1 [file DataSheet1.ZIP › Supplementary files/Figure S3.jpg]
